# Assembly and coherent control of a register of nuclear spin qubits

**DOI:** 10.1038/s41467-022-29977-z

**Published:** 2022-05-19

**Authors:** Katrina Barnes, Peter Battaglino, Benjamin J. Bloom, Kayleigh Cassella, Robin Coxe, Nicole Crisosto, Jonathan P. King, Stanimir S. Kondov, Krish Kotru, Stuart C. Larsen, Joseph Lauigan, Brian J. Lester, Mickey McDonald, Eli Megidish, Sandeep Narayanaswami, Ciro Nishiguchi, Remy Notermans, Lucas S. Peng, Albert Ryou, Tsung-Yao Wu, Michael Yarwood

**Affiliations:** Atom Computing, Inc., Berkeley, CA 94710 USA

**Keywords:** Qubits, Atomic and molecular interactions with photons

## Abstract

The generation of a register of highly coherent, but independent, qubits is a prerequisite to performing universal quantum computation. Here we introduce a qubit encoded in two nuclear spin states of a single ^87^Sr atom and demonstrate coherence approaching the minute-scale within an assembled register of individually-controlled qubits. While other systems have shown impressive coherence times through some combination of shielding, careful trapping, global operations, and dynamical decoupling, we achieve comparable coherence times while individually driving multiple qubits in parallel. We highlight that even with simultaneous manipulation of multiple qubits within the register, we observe coherence in excess of 10^5^ times the current length of the operations, with ${T}_{2}^{{{{{\mathrm{echo}}}}}}=\left(40\pm 7\right)$ seconds. We anticipate that nuclear spin qubits will combine readily with the technical advances that have led to larger arrays of individually trapped neutral atoms and high-fidelity entangling operations, thus accelerating the realization of intermediate-scale quantum information processors.

## Abstract (web summary)

In large qubit registers, long coherence times and individual qubit control are difficult to achieve at the same time. Here, the authors assemble a 2D register of qubits in an array of fermionic alkaline-earth atoms, where tailored pulses can be applied to subsets of individual qubits in parallel.

## Introduction

The proposed use of nuclear spins to encode and store quantum information has a long history owing to their isolation from undesired interactions with the environment^1,2^. The difficulty of reliably measuring nuclear spin states has historically limited the adoption of nuclear spin qubits outside of ensemble quantum computing demonstrations^3,4^. As the control and detection of individual quantum systems have advanced, the use of the nuclear spin degree of freedom has consistently shown favorable coherence when compared to electronic spin degrees of freedom^5–8^. However, these demonstrations have either relied on a global control architecture or tailored interactions with neighboring quantum systems, both of which impede scaling to large numbers of qubits using current technology.

Individually trapped neutral atoms in optical tweezers are a promising platform for the study of quantum many-body systems, combining exquisite control over the full quantum state of individual atoms with the ability to efficiently scale to larger numbers of atoms with modest overhead and minimal reduction in the per-atom fidelity^9,10^. Until recently, optical tweezer systems primarily used alkali metal atoms, which have favorable level structures for rapid loading and cooling of the atoms, along with the ground-state hyperfine structure that enables the manipulation of metastable spin states via microwave transitions^11–15^. However, optical tweezer technology is agnostic to the specific atom chosen. Recent work has demonstrated the ability to use the same platform for trapping alkaline-earth atoms, which have attractive properties for the storage and coherent manipulation of quantum information, as well as for cooling, state preparation, and measurement of the internal state of the atoms^16–22^. Fermionic isotopes of alkaline earth atoms retain these same favorable properties while also possessing a non-zero nuclear spin, which can be leveraged for defining a qubit manifold.

In this manuscript, we introduce Phoenix, a system for assembling a register of highly coherent qubits encoded in the nuclear spin degree of freedom of atoms with a closed-shell *S*-orbital. The system is an evolution of recent alkali-based programmable quantum simulators^9,10,23–25^, but, importantly, Phoenix is able to apply tailored pulses to subsets of individual qubits in parallel—a crucial feature for gate-based quantum computation. In particular, we trap individual ^87^Sr atoms in an array of optical tweezers, prepare a uniformly filled register of spin-polarized atoms, then individually manipulate, and read out the spin state of the qubits. We highlight the coherence of quantum information encoded in the ground-state nuclear spin manifold of ^87^Sr atoms, demonstrating the advantages of this qubit encoding and therefore the promise of this platform for quantum information storage.

## Results

**Fig. 1:**
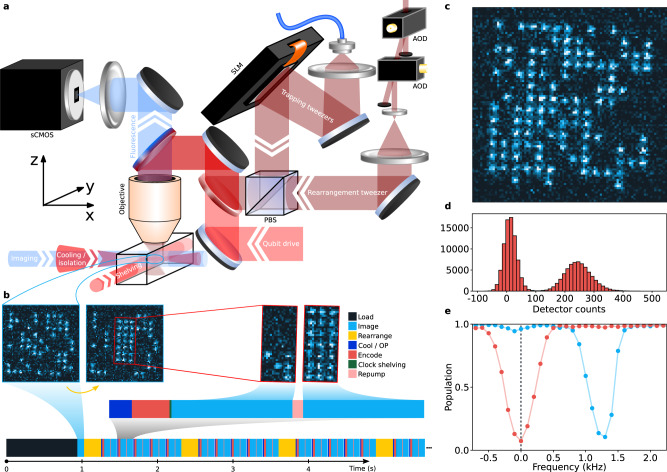
Machine to assemble a register of nuclear spin qubits. **a** Schematic of the primary components of the optical tweezer system that trap, rearrange, manipulate, and read out the state of the nuclear spin qubits. The static trap array is generated using a spatial light modulator (SLM), and rearrangement light is steered using a pair of crossed AODs and combined with the trap light on a polarizing beam splitter (PBS). The qubit drive light is combined with both of these beams using a dichroic mirror. All of these beams are directed into the microscope objective using another dichroic mirror that transmits the collected atom fluorescence signal (461 nm) to the imaging system that forms an image on the commercial sCMOS camera. **b** Experimental sequence timing. After loading the trap array, and again after several experiment repetitions (the exact number is varied based on the probability to lose an atom in each experiment), we perform rearrangement to fully fill the computational array, forming a register of qubits. **c** Single fluorescence image demonstrating our ability to load over 100 atoms, which could be rearranged into a larger computational array than was used for this work. **d** Histogram of photon counts collected from a single site, summed across 6750 images and all 21 qubits in the register. The two peaks indicate the presence or absence of an atom that fluoresces on that site. **e** Clock-state shelving spectroscopy was taken when preparing the qubits in either $\left|\downarrow \right\rangle$ (red) or $\left|\uparrow \right\rangle$ (blue). By driving a transition at the frequency of the black dashed line, the spin selective readout can be performed.

Our array of optical tweezer trapping potentials is generated holographically, as shown in Fig. 1a, using a liquid crystal on silicon spatial light modulator (SLM), which imprints a spatially varying phase pattern on the beam before it is focused by a high-numerical-aperture microscope objective^26,27^. We are able to programmatically define the trap geometry (array size, shape, spacing, and relative depth) using the SLM, which gives us the flexibility to rapidly change the computational array geometry depending on what is required for a particular computation. For this work, we define a rectangular trap array with 110 total trapping sites (10 × 11 sites) with a 4-μm separation between each trap center. The trapped atoms can be used to perform many tens of state-preparation, circuit, and measurement cycles before reloading the trap array. Figure 1b gives an example of one such cycle. The choice of array size is somewhat arbitrary; while the 10 × 11 array allows us to load ~50 ^87^Sr atoms on average, we show in Fig. 1c that a larger 14 × 14 array can trap over 100 atoms.

Atoms are initially loaded into the optical tweezers after several laser cooling stages. We additionally perform Sisyphus cooling on the trapped atoms to bring them near the bottom of the optical tweezer potential^16,17,28^. The initial loading of traps is stochastic, so we define a subset of the trap array to be the computational array, which will define our register of qubits ^11,29^. The computational array comprises 21 qubits in a fully filled 7 × 3 sub-array of traps. We uniformly fill the computational array using a dynamic optical tweezer, pictured in Fig. 1a, that rearranges the atoms by dragging atoms from filled sites and placing them into empty sites^15,30,31^. Importantly, determining if a site is occupied is accomplished by illuminating the array with light that is near-resonance with the ^1^*S*_0_ → ^1^*P*_1_ transition, while simultaneously cooling the atoms with light near-resonance with the ^1^*S*_0_ → ^3^*P*_1_ transition^19^. The resulting atomic fluorescence is then imaged onto a scientific complementary metal-oxide-semiconductor camera (see “Methods”).

Images such as the one shown in Fig. 1c, combined with thresholding derived from histograms classifying counts collected per qubit, as shown in Fig. 1d, reveal the spatial locations of strontium atoms in the ^1^*S*_0_ manifold and whether or not they are fluorescing. However, these images do not distinguish between atoms in different nuclear spin sublevels. To detect the nuclear spin state, we use the long-lived ^3^*P*_0_ manifold (typically used by state-of-the-art optical atomic clocks) to shelve the population that we do not want to detect^20–22^. For example, by driving a *π* rotation between the ${\left.\right|}^{1}{S}_{0},\,F=9/2,\,{m}_{F}=-9/2\left.\right\rangle$ nuclear-spin ground state and ${\left.\right|}^{3}{P}_{0},\,F=9/2,\,{m}_{F}=-9/2\left.\right\rangle$ upper clock state, we “shelve” any population in the nuclear spin state into the clock state (see the red measurements in Fig. 1e), such that a subsequent fluorescence image will ideally only capture photons scattered from atoms that were in another nuclear spin state. However, the lifetime of the shelved state is reduced from its natural lifetime of thousands of seconds down to of order 1 s due to the large intensity of the trap light causing Raman scattering within the triplet spin manifolds, which leads to further decay to the ground state manifold^20,21,32^. This does not prevent the efficient detection of the nuclear spin state. In “Methods,” we describe how we compensate our measurements for these readout errors. In the future, a combination of more sensitive detectors, higher collection efficiency objectives, and lower scattering readout traps will ensure high-fidelity single-shot readout, for example, to implement mid-circuit measurements for quantum error correction protocols. Alternatively, the use of a state-selective transition to induce fluorescence without shelving would sidestep this issue entirely (see “Methods”).

**Fig. 2:**
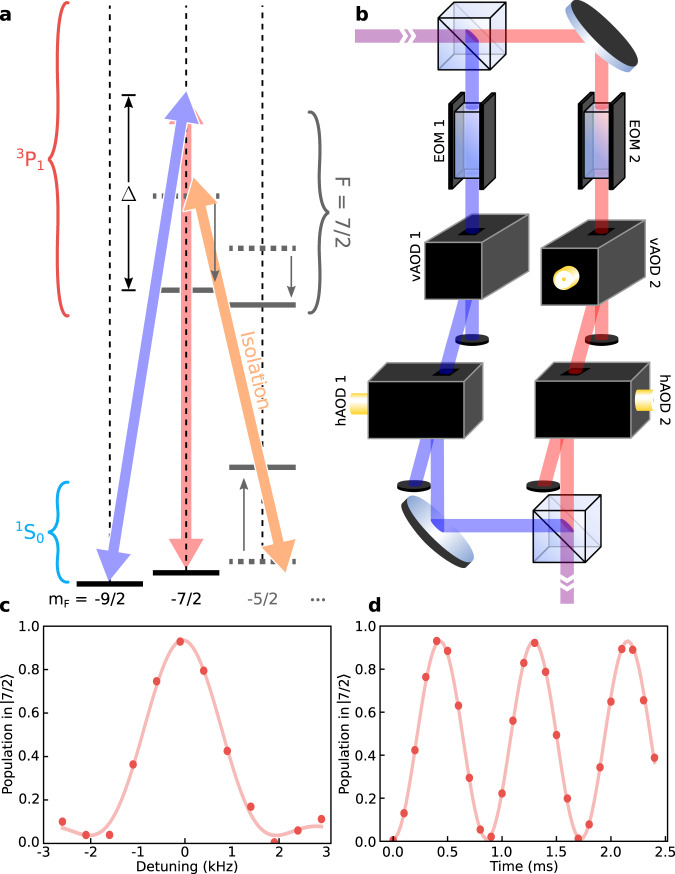
Isolating and manipulating a nuclear spin qubit. **a** Simplified level diagram for ^87^Sr showing the nuclear spin qubit states $\left|\downarrow \right\rangle \equiv {\left.\right|}^{1}{S}_{0},\,F=9/2,\,{m}_{F}=-9/2\left.\right\rangle$ and $\left|\uparrow \right\rangle \equiv {\left.\right|}^{1}{S}_{0},\,F=9/2,\,{m}_{F}=-7/2\left.\right\rangle$, and how they are coupled via a two-photon Raman transition with two orthogonally polarized drive beams detuned by an amount Δ from the ^3^*P*_1_, *F* = 7/2 manifold. Also indicated is the Stark-shift beam, which is used to isolate the qubit manifold from the rest of the *I* = 9/2 nuclear spin manifold by shifting the leakage transition (see main text) out of resonance with the two-photon drive. **b** A schematic showing the preparation of the two drive beams with one electro-optic modulator (EOM) and two crossed acousto-optic deflectors (AODs) in each beam path. The EOMs are used for global, fast pulse shaping, while the pair of vertical and horizontal AODs (vAOD and hAOD, respectively) are used to adjust the phase and amplitude of each beam on a site-by-site basis. **c** Nuclear spin qubit resonance measured by scanning the modulation frequency of EOM1 while driving for a fixed duration of 446 μs. Note that the qubit frequency is set by the applied 11-Gauss magnetic field, which defines the quantization axis. **d** Rabi flops on a nuclear spin qubit, taken by setting the qubit frequency (which defines the exact drive frequency of EOM1) to 2.1 kHz and scanning the length of the drive pulse. Each data point comprises >2900 counts and is plotted with error bars representing the standard error of the mean (typically smaller than the point markers).

As depicted in Fig. 2a, we define our qubit manifold as the two lowest-lying nuclear spin states in a positive magnetic field: $\left|\downarrow \right\rangle \equiv {\left.\right|}^{1}{S}_{0},F=9/2,{m}_{F}=-9/2\left.\right\rangle$ and $\left|\uparrow \right\rangle \equiv {\left.\right|}^{1}{S}_{0},F=9/2,{m}_{F}=-7/2\left.\right\rangle$. However, for any reasonably small magnetic field, the transition frequency between our qubit states will be degenerate with the leakage transition from $\left|\uparrow \right\rangle$ to $\left|L\right\rangle \equiv {\left.\right|}^{1}{S}_{0},\,F=9/2,\,{m}_{F}=-5/2\left.\right\rangle$ (and with the subsequent transitions that drive qubits further out of the qubit manifold and into higher nuclear-spin ground states). To isolate the qubit manifold from other nuclear spin states, we apply a strong “Stark-shift beam” (orange arrow in Fig. 2a) that is nearly resonant with the $\left|L\right\rangle \to {\left.\right|}^{3}{P}_{1},\,F=7/2,\,{m}_{F}=-7/2\left.\right\rangle$ transition, which shifts this nuclear spin state (and thus the primary leakage transition) out of resonance with any drive that performs rotations within the qubit manifold. Because no population ends up in the $\left|L\right\rangle$ state and the polarization of the beam is such that the nearest transition from the qubit states allowed by electric dipole selection rules is far detuned (over 1 GHz), photon scattering from the qubit states due to this beam can be suppressed to a rate of ~1 photon per second.

Site-resolved qubit state manipulations are enabled by the “Qubit drive” beam path depicted in Fig. 1a, which includes two beams for each atom being addressed that are projected through the same microscope objective. The intensity profile of each addressing beam closely resembles that of an Airy disk, with the location of the second intensity minimum approximately coinciding with the qubit spacing. We estimate the average cross-talk of the local drive beams to neighboring atoms is $< \left(0.5\pm 0.1\right)$% (see “Methods”). The beams are detuned from the ^3^*P*_1_ manifold of states in order to drive two-photon Raman transitions between the nuclear-spin ground states. As shown in Fig. 2b, the beams share a common laser source, with each one being spatially divided and steered to the atoms using a pair of crossed acousto-optic deflectors (AODs). We achieve full amplitude and phase control over the two-photon transition at each site in the array by adjusting the radio-frequency tones driving the AODs that correspond to addressing each qubit. Importantly, the AODs are oriented such that the detuning between the beams is both finite and constant across the array of sites—this offers a separate degree of freedom for actuating the two-photon coupling, while also ensuring that atoms can be driven in parallel. On Phoenix, we can apply operations in parallel only on atoms in a single column (or row) and serially apply drives to qubits in separate columns (or rows). Specifically for all data presented here, the register of 21 qubits is addressed by column: this means that up to seven qubits are driven simultaneously and all 21 can be addressed with three groups of pulses. This approach ensures that we can have full control over the operation applied to each qubit, independent of the drive applied to any other qubits. Turning on the two-photon drive is accomplished by driving a pair of electro-optic phase modulators (EOMs), one in each of the two addressing beams (see Fig. 2b). The first-order sidebands of the two EOMs drive the primary Raman process. The EOMs enable fast pulse shaping and rapid adjustments to the intermediate state detuning.

Adjusting the drive frequency of either EOM effectively tunes the relative frequency of the two beams, which we can use to find the qubit transition, as seen in Fig. 2c. By varying the length of the EOM drive, we then observe coherent Rabi oscillations between the two qubit states (see Fig. 2d), demonstrating that the qubit manifold is well isolated. Tuning the drive time to 223 μs realizes a *π*/2 pulse, which we define to be the $\hat{x}$-axis of the Bloch sphere. We note that our choice of Rabi frequency of 1.16 kHz must be significantly smaller than the isolation of the qubit manifold provided by the Stark-shift beam (orange arrow in the level diagram of Fig. 2a), which is primarily limited by laser power and polarization purity.

**Fig. 3:**
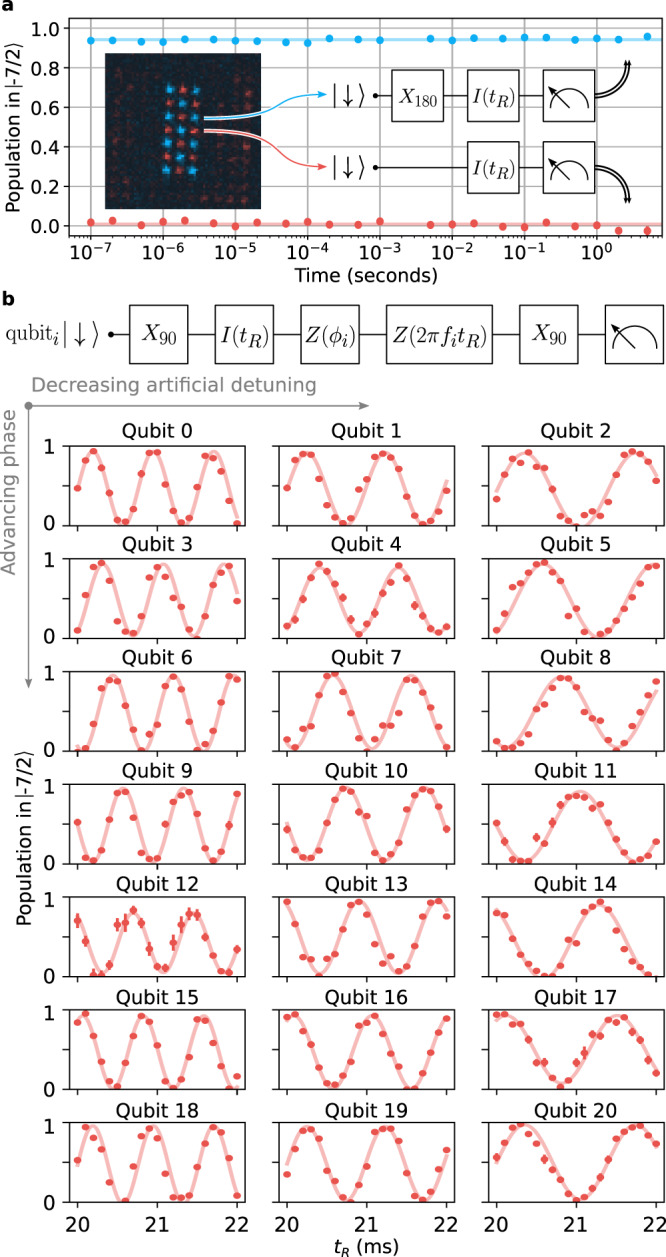
Site-resolved manipulation within the register of qubits. **a** A measurement to bound the relaxation time (*T*_1_) between the qubit states, taken in a single run by selectively rotating a subset of the qubits (indicated in the inset) to the $\left|\uparrow \right\rangle$ state before a variable hold time. Each data point comprises >600 counts. **b** A demonstration of Ramsey oscillations on individual qubits, where each qubit is given a unique combination of static phase offset *ϕ*_*i*_ and artificial detuning *f*_*i*_ (where *f*_*i*_ is the rate of phase accumulation used to set the phase of the final *π*/2 pulse), as specified by the circuit diagram. Solid lines are sinusoidal curves with phase offset and artificial detuning fixed to their programmed values. Each point comprises about 200 counts. All data points have error bars representing the standard error of the mean, but in most cases these are smaller than the point markers.

With the ability to site-selectively drive individual qubits, we attempt to bound the spin relaxation timescale *T*_1_ within the qubit manifold in a single experiment by performing standard state preparation on the full register of qubits and an additional *π* rotation on 11 of the qubits (using a checkerboard pattern). After preparing 10 qubits in $\left|\downarrow \right\rangle$ and 11 qubits in $\left|\uparrow \right\rangle$, we wait for a variable hold time to observe relaxation in the spin states over time. As can be seen in Fig. 3a, the depolarization timescale is significantly longer than 10 s—consistent with no depolarization on this timescale. While this demonstrates that the qubit states are not depolarizing on timescales where we begin to be limited by the vacuum lifetime of the traps (approximately 60 s), we emphasize that increasing the vacuum lifetime beyond 400 s is routine in room-temperature atomic physics laboratories^19^, and extending that further is likely possible by placing such trapping regions inside a cryogenic environment^33^.

We now turn our attention to experiments that are sensitive to the phase coherence of the qubit manifold by encoding a superposition state and reading out that superposition after some delay^34^. The canonical experiment to demonstrate the coherence of a qubit is the Ramsey sequence, which consists of two *π*/2 rotations separated by a varying length of time, *t*_R_, as depicted in the circuit diagram in Fig. 3b. In these experiments, we vary the phase of the second *π*/2 pulse linearly in *t*_R_ to apply an “artificial detuning” that creates an oscillatory signal. Dephasing in the qubit manifold would generally reduce the contrast of these oscillations.

In a first Ramsey experiment, we emphasize our ability to individually drive qubits within the computational array in parallel. In Fig. 3b, we perform a Ramsey sequence with three unique artificial detunings *f*_*i*_ on each column of seven qubits (*f*_*i*_ ∈ {0.7, 1, 1.3} kHz). Furthermore, we apply seven unique phase offsets *ϕ*_*i*_, one for each row of three qubits (−*π* ≤ *ϕ*_*i*_ ≤ *π*). As a result, every individual qubit should have a different Ramsey oscillation. Note again that all seven qubits in a single column were driven simultaneously in this experiment. The solid lines are sinusoidal fits with frequency and phase offset fixed to their programmed values, with only amplitude and vertical offset left as free parameters. Their agreement with the data demonstrates our ability to fully control phase and frequency for every qubit.

**Fig. 4:**
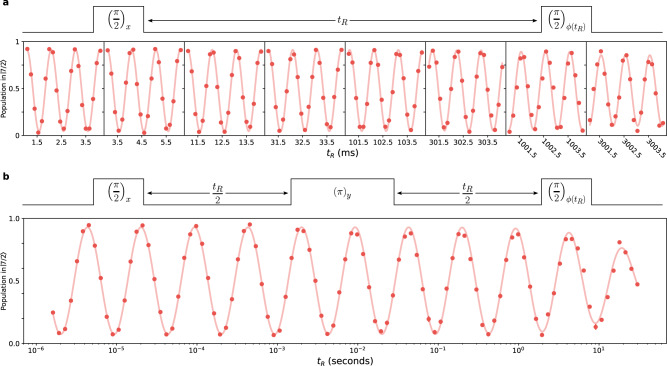
Coherence of nuclear spin qubits. **a** Array-averaged oscillations taken in a Ramsey experiment with variable hold time used to bound the coherence time ${T}_{2}^{\star }\gg 3$ s. The plotted exponentially decaying sinusoid is a fit to the data and gives a dephasing time ${T}_{2}^{\star }=\left(21\pm 7\right)$ s. Note that the *x*-axis is split into discrete time windows of 3 ms each, spaced at exponentially increasing time offsets out to over 3 s. The ability to see coherent oscillations when averaging the signal across the entire array highlights the uniformity of the qubit frequency across the array, but the slight reduction in contrast likely indicates slight miscalibrations of the pulse area used to encode and read out the phase of each qubit. Each data point comprises >1700 counts. **b** Array-averaged Ramsey-echo coherence measurement taken at logarithmically spaced Ramsey evolution times *t*_R_ (as indicated in the pulse diagram, half of the evolution time happens between the first *π*/2 pulse and the echo *π* pulse, while the remainder happens in between the echo *π* pulse and the final *π*/2 pulse). By setting the phase of the final pulse to advance proportionally with $\log ({t}_{{{{{{\rm{R}}}}}}})$, we can plot these measurements on a continuous semi-log plot and see oscillations that appear sinusoidal. The plotted curve shows an exponentially decaying sinusoid (with logarithmically advancing phase) fitted to the data, which estimates a dephasing rate ${T}_{2}^{{{\mbox{echo}}}}=\left(40\pm 7\right)$ s. Each data point comprises >1000 counts and includes error bars representing the standard error of the mean, but in most cases these are smaller than the point markers.

The Ramsey oscillations are expected to decay on an exponential timescale as the qubits dephase. To measure the decay, we take similar snapshots of the Ramsey oscillations (same artificial detuning, time span, and point spacing), but with an exponentially increasing time offset. To more clearly display these data, Fig. 4a uses a split *x*-axis, where we cut out the large segments of *t*_R_ that have no data present. In contrast to Fig. 3b, here we present the data averaged over all 21 qubits, which is possible due to the uniformity of the qubit frequency and response to the drive light across the computational array. It is immediately clear that the contrast remains very large out to times exceeding 3 s. The solid curve is a simultaneous fit to all the measurements, highlighting the phase stability of the Ramsey oscillations. This indicates not only that changes to the relative phase for the drive beams are small on the seconds timescale, but also that the qubit frequency is not drifting significantly on the timescale of the entire experiment (taken in pieces over the course of 2 days). By fixing the frequency, phase, and initial amplitude for the fit, we can extrapolate an estimate of the dephasing timescale that is ${T}_{2}^{\star }=\left(21\pm 7\right)$ s. Even without extrapolating to times longer than the plotted data, we can safely bound the dephasing as ${T}_{2}^{\star }\gg 3$ s.

In an attempt to more directly show the magnitude of the coherence of the nuclear spin qubits, we perform a modified Ramsey echo experiment, where we add a single echo pulse (a *π* rotation about the $\hat{y}$-axis of the Bloch sphere) in between the two *π*/2 pulses, as depicted in Fig. 4b, and perform measurements out to 30 s hold times. A typical Ramsey echo experiment adjusts the phase of the final *π*/2 pulse in the same way that we did in the earlier Ramsey experiments. Here we opt to explicitly adjust the phase logarithmically in *t*_R_ to give familiar sinusoidal oscillations when plotted on a semilog plot. The addition of the echo pulse to this sequence makes us less sensitive to any small deviation of the qubit frequency, which is important because the rate of phase accumulation at *t*_R_ = 30 s is slow enough that even a deviation of 25 mHz (~10 ppm of the qubit frequency) would significantly alter the period of the oscillations compared to shorter *t*_R_ values. To extract an estimate of the decay constant, we fit the Ramsey echo measurements using a sinusoidal model given by the equation $y=b+a{e}^{-t/\tau }\sin (\phi +2\pi n\log ({t}_{{{{{{\rm{R}}}}}}}))$, where *y* is a measurement, *τ* is the decay constant, *ϕ* is a phase offset, *a* is an amplitude, *b* is a vertical offset, *n* is the number of oscillations per decade of time points, and *t*_R_ is the independent variable. By fixing the values of *n* and *ϕ* (set by fitting earlier oscillations without a decay term), we obtain an estimate of the decay time constant of $\left(40\pm 7\right)$ s. While the array-averaged data have been fit to estimate the overall coherence time of each qubit, the fits are consistent with the fitted values from individual site data.

## Discussion

Future work is underway to optimize the operational parameters of this system, as well as to explore methods to mitigate undesired scattering that currently limits the performance of our system. Specifically, we anticipate that the use of larger magnetic fields and different state detunings will improve undesired scattering rates from the stark shifting beam during qubit manipulations. Additionally, we can take advantage of pulse shaping and composite pulses to improve the uniformity of rotational area for each atom in the array, which currently limits the contrast of the array-averaged oscillations presented. Separately, the single-shot readout fidelity is currently limited by Raman scattering out of the ^3^*P*_0_ manifold during the readout process, which can be further optimized with faster imaging, or by direct spin-resolved excitation on the ^3^*P*_1_ transition (see “Methods”).

In conclusion, we have demonstrated the encoding of a qubit in the nuclear spin degree of freedom of individually trapped neutral atoms. Furthermore, we have introduced a platform that can assemble an individually-addressable register of nuclear spin qubits and is compatible with increased computational array sizes, as well as reduced gate operation times. Future work in both strontium and other elements with similar level structures will tackle increasing the qubit coherence time via a combination of lower noise local oscillators as well as magnetic shielding while increasing the driven Rabi rates by multiple orders of magnitude with the goal of reaching system coherence times that are 10^8^ times the length of the individual gates. The ability to individually encode, manipulate, and read out these qubits is an important first step in demonstrating this platform as a leading contender for the realization of a universal quantum computer.

## Methods

### Atom loading, cooling, state preparation, and measurement

The process of initializing the qubit starts with producing a strontium atomic beam in an ultra-high vacuum (UHV) system. The atomic beam is slowed by optical forces from a Zeeman slower and 2D magneto-optical trap (MOT). A second 2D MOT then directs the atoms toward a UHV glass cell (~10^−11^ Torr), where a 3D MOT cools an ensemble of atoms to millikelvin-scale temperatures^35^. These three cooling stages all operate on the ^1^*S*_0_ → ^1^*P*_1_ manifold transitions at 461 nm. The atoms are then further cooled by a second 3D MOT, overlapped with the 461-nm MOT. This second MOT operates on the narrow ^1^*S*_0_ → ^3^*P*_1_ intercombination line (natural linewidth of 7.4 kHz) at 689 nm, with laser beams that are frequency modulated to create a sawtooth-wave adiabatic passage (SWAP) MOT. The modulated light efficiently captures hotter atoms from the blue MOT^16,28^. When this frequency modulation stops, the narrow linewidth of the 689-nm transition is fully utilized to cool the atoms into co-located optical tweezer traps.

The tweezers operate at *λ* = 813.4 nm, the magic wavelength for the optical clock transition from ${\left.\right|}^{1}{S}_{0},\,F=9/2,\,{m}_{F}=-9/2\left.\right\rangle$ to ${\left.\right|}^{3}{P}_{0},\,F=9/2,\,{m}_{F}=-9/2\left.\right\rangle$ (Fig. 1e shows a frequency scan over this transition). We have measured radial trap frequencies of 95 kHz and trap depths of ~6 MHz. The traps are loaded stochastically from the MOT cooling stages with, in some cases, >1 atom. To reduce the per-trap atom number to 0 or 1, we apply a photoassociation pulse^29^ at 461 nm such that pairs of atoms are ejected from the traps. For each trap loading cycle, we subsequently run many sequences of atom rearrangement, state-preparation, gates, and measurements (as described below; also see the sequence diagram in Fig. 1b) before reloading from MOTs becomes necessary due to atom loss during imaging or via background gas collisions. Our current vacuum-limited atom lifetime is ~60 s and can be readily extended with improved pumping speeds and cryogenics.

Our choice of tweezer array size is mainly limited by the maximum trap depth which can be achieved with a finite amount of laser power. Operating at lower tweezer depth results in two deleterious effects. First, the maximum scattering rate which can be achieved is necessarily lower in shallower traps, since larger scattering rates increase the probability that an atom will escape from a weakly confining potential during imaging. We have found that the maximum achievable scattering rate supporting a fixed survival probability between consecutive images scales approximately linearly with tweezer trap depth, meaning that shallower tweezers require longer camera exposure times to achieve acceptable SNR. Our choice of demonstrating coherent control in a 10 × 11 tweezer array supporting a 21 qubit register throughout our experimental cycle is a compromise between the desire for a large array size and the necessity of using short exposure times to increase the data acquisition rate. Second, loading into optical tweezers is less efficient at lower trap depth. In order for an atom to fall into the potential defined by an optical tweezer with high probability, the trap depth must exceed the equilibrium temperature of atoms during their final stage of MOT cooling. The equilibrium temperature for atoms in our final stage of cooling is likely limited by the quality of our narrow-line 689 nm laser lock, and operating with a 110 tweezer array ensures that we load enough atoms to fill (and refill multiple times during the experimental cycle) a 21 qubit register, despite not quite reaching 50% loading efficiency.

After atom loading, which typically occurs in <1 s, the individual atoms are rearranged into a uniformly filled grid near the center of the trap array using a dynamic optical tweezer controlled by the pair of crossed AODs pictured in Fig. 1a^30^. The atoms are then optically cooled to lower motional states of the trap using the Sisyphus cooling mechanism^17^. This cooling frequency is red-detuned from the ${\left.\right|}^{1}{S}_{0},\,F=9/2,\,{m}_{F}=-9/2\left.\right\rangle \to {\left.\right|}^{3}{P}_{1},\,F=11/2,\,{m}_{F}=-11/2\left.\right\rangle$ transition with *σ*^−^ polarization, which places this process in the attractor regime of Sisyphus cooling. Efficient cooling with a global beam is best achieved when operating with uniform-intensity traps, since variations in trap-induced light shifts across the array remain small compared to the linewidth of the cooling light. The atom temperature at the end of this stage is ~4 μK. After and during cooling, we also optically pump the atoms using light tuned near the ${\left.\right|}^{1}{S}_{0}\left.\right\rangle \to {\left.\right|}^{3}{P}_{1},\,F=9/2\left.\right\rangle$ transitions with *σ*^−^ polarization. This choice of polarization and laser detuning leaves the ${\left.\right|}^{1}{S}_{0},\,{m}_{F}=-9/2\left.\right\rangle$ state dark to the excitation light^36^.

We then apply a sequence of gates to the qubits, as described in the main text, before performing a projective measurement. The measurement is performed by applying a global pulse of resonant light at 461 nm that induces atom fluorescence on the ${\left.\right|}^{1}{S}_{0}\left.\right\rangle \to {\left.\right|}^{1}{P}_{1}\left.\right\rangle$ transition, as described in the main text. To read out the individual nuclear spin states, we shelve one of them into a metastable clock state in the ^3^*P*_0_ manifold prior to applying a first imaging pulse. In order to monitor and correct for atom loss, we post-select by repumping the shelved atoms to the ground states and then applying a second imaging pulse. The broad linewidth of the imaging transition not only allows for rapid photon scattering for detection but also causes detrimental heating of the trapped atoms that can lead to atom loss. To avoid dislodging atoms from their respective tweezers, we counteract this heating by applying Sisyphus cooling simultaneously^19^, in addition to carefully setting the intensity and frequency of the imaging light.

### Trap array generation and flattening

The tweezer traps are produced at the focal plane of a custom high-NA (0.65) microscope objective with a 300-micron diffraction-limited field of view. A phase mask is imprinted on the trap light by a spatial light modulator (SLM) and optically relayed onto the back focal plane of this objective, generating nearly arbitrary and reconfigurable two-dimensional arrays of optical tweezers. The spatial phase imparted by the SLM is optically Fourier transformed by the microscope objective to create a grid of focused spots. We use the weighted Gerchberg–Saxton algorithm to calculate the appropriate phase mask for the SLM^37^.

### Rearrangement

Rearrangement is performed using a single focused beam derived from the same Ti:Sapphire laser source used to create the static computing traps. This beam is steered using a pair of crossed AODs, which are driven by RF waveforms generated by custom FPGA hardware. In order to rearrange atoms into a desired target pattern, an image is first taken which establishes the locations of initially occupied sites. This image is used to calculate a set of moves to fill target sites from the initially occupied sites according to the compression algorithm^38^. Before performing the calculated sequence of moves, the depth of the static computing traps is lowered to ~20% of their initial value. Moves are then performed in three steps: (1) ramp up the intensity of the rearrangement beam, (2) translate the rearrangement beam from the initial site to the target site using linear frequency chirps on the AODs, and (3) ramp down the intensity of the rearrangement beam.

### POVM measurement correction

As discussed in the main text, our qubit readout scheme involves shelving of the $\left|\downarrow \right\rangle$ state population in a metastable level within the (5s5p) ^3^*P*_0_ manifold. Population in this manifold experiences Raman scattering due to 813-nm trapping light, and ultimately decays into the ground state by way of the (5s5p) ^3^*P*_1_ manifold^32^. As a result, the $\left|\downarrow \right\rangle$ state is incorrectly measured as $\left|\uparrow \right\rangle$ with some probability *q*. We correct for such errors in reading out the $\left|\downarrow \right\rangle$ qubit level using the formalism of positive-operator valued measure (POVM) corrections, which we briefly summarize here^39^. In this approach, the POVM **M**^ideal^ for an ideal single-qubit projective measurement in the computational basis is

1 $${M}_{1}=\left[\begin{array}{cc}1&0\\ 0&0\end{array}\right],\,\,{M}_{2}=\left[\begin{array}{cc}0&0\\ 0&1\end{array}\right].$$

For a non-ideal measurement, the POVM becomes **M**^exp^ = Λ**M**^ideal^, where Λ is an invertible matrix whose elements comprise the conditional measurement probabilities for each qubit state. From this, one finds the intuitive result that the non-ideal POVM is given as

2 $${{{{{{{{\bf{M}}}}}}}}}_{1,{{{{\mathrm{exp}}}}}}=\left[\begin{array}{ll}1-p&0\\ 0&q\end{array}\right],\,\,{{{{{{{{\bf{M}}}}}}}}}_{2,{{{{\mathrm{exp}}}}}}=\left[\begin{array}{ll}p&0\\ 0&1-q\end{array}\right],$$

where, in a manner analogous to *q*, *p* is the probability that $\left|\uparrow \right\rangle$ is incorrectly measured in the $\left|\downarrow \right\rangle$ state. The measurement correction matrix is then

3 $${{{\Lambda }}}^{-1}=\frac{1}{1-p-q}\left[\begin{array}{ll}1-q&-q\\ -p&1-p\end{array}\right],$$

such that the measured vector of probabilities *P*_exp_ (i.e., [*m*_exp_, 1 − *m*_exp_], with each element corresponding to a qubit level) can be corrected with the equation *P*_corr_ = Λ^−1^*P*_exp_. Using our definition of Λ^−1^, we then correct our raw measurements *m*_exp_ as follows:

4 $${m}_{{{{{\mathrm{corr}}}}}}=\frac{{m}_{{{{{\mathrm{exp}}}}}}-q}{1-p-q}$$

Additionally, error bars for a particular measurement are corrected by using this equation to propagate the uncertainties in *m*_exp_ and *q*.

**Fig. 5:**
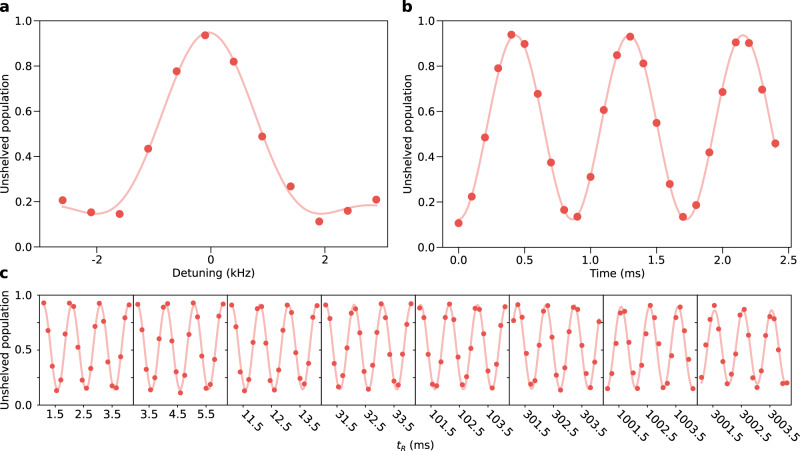
Selected measurements shown without POVM corrections. Uncorrected measurements of **a** the nuclear-spin qubit resonance originally shown in Fig. 2c, **b** Rabi flopping of the nuclear-spin qubit originally shown in Fig. 2d, and **c** Ramsey oscillations originally shown in Fig. 4a. Solid lines represent fits that use the same underlying models as in the main text to fit these uncorrected data points. Information regarding error bars and counts-per-point are provided in the main text.

Typically, one would run dedicated experiments to measure the probabilities *p* and *q* and construct the POVM. In our system, however, atoms trapped outside the computational array are used for in-situ measurements of the probability *q*, as they are prepared in the $\left|\downarrow \right\rangle$ state with high efficiency (see, for example, Fig. 1e) and remain undriven. In fact, our measurement of *q* is updated from one point to the next during an experimental parameter scan, since the undriven atoms are always read out. To calculate *q* for a set of experimental shots, we start by summing the bright counts from all the undriven atoms, with each count conditioned on the corresponding atom remaining trapped throughout the measurement. This sum is then normalized by the total counts from all the undriven atoms, conditioned in the same way as before. We also conservatively set the probability *p* to 0, as this amounts to no measurement correction for the $\left|\uparrow \right\rangle$ qubit state. In summary, our procedure amounts to using atoms outside the computational array to find an average value of *q* for each condition of a parameter sweep and then applying that value to atoms within the computational array in post-processing. Since these corrections have been applied to the measurements shown in the main text, in Fig. 5 we provide three representative examples of uncorrected measurements.

### Readout error mitigation

Raman scattering from ^3^*P*_0_ can be reduced by minimizing the amount of time atoms spend in the clock state before imaging. One strategy for achieving this, recently implemented in ^88^Sr^40^, is to remove the ground state population via a pulse of resonant 461 nm light immediately after the clock pulse is performed. This resulted in 99% readout fidelity but came at the cost of requiring refilling of the array after each measurement, which is incompatible with our engineering requirements.

Another option is to mitigate scattering from ^3^P_0_ by lowering trap depth and shortening imaging exposure time via faster ^1^S_0_ → ^1^P_1_ scattering rates^19^, albeit at the cost of lower survival probability between consecutive images. This strategy was used to create the data presented in Fig. 1d, e, resulting in a probability of correctly distinguishing whether or not a tweezer is occupied of 0.9997(2), a probability of atom loss between consecutive images of 0.045(1), and a probability of correctly distinguishing whether an atom is in $\left|\uparrow \right\rangle$ or $\left|\downarrow \right\rangle$ of approximately 0.927. Future measurements can push non-POVM-corrected fidelity higher (and atom loss lower) by collecting photons more efficiently, e.g., by increasing microscope numerical aperture, by lowering readout noise, e.g., by correcting aberrations of the point spread function to focus light more efficiently onto a single pixel, or by better balancing the probabilities of atom survival, correct state detection assignment, and successful histogram-based tweezer occupation readout, e.g., by trading fewer photons (lower tweezer occupation readout fidelity) for less Raman scattering from ^3^P_0_ (better state-detection fidelity). Another approach involves using the narrow ^3^*P*_1_ intercombination line to sequentially image atoms in each qubit level (eliminating the need for shelving in the clock state altogether), although the smaller scattering rate in this case would lower the readout speed^41^. Finally, this error is only a limitation when single-shot measurements are required (e.g., for feed-forward techniques or for shot-to-shot correlations of the measurement outcomes).

### Fitting of data

For all fitted data presented, we perform a weighted least-squares optimization with the indicated functional form. Any fixed parameters for the fits are indicated in the text and are set with independent measurements (e.g., the initial amplitude of the oscillations in Fig. 4) or based upon the defined experimental sequence (e.g., the phase offsets that are programmatically defined for Fig. 3b). All fits reported in the main text are performed after applying the POVM correction to the data based on the shelving probability of the atoms outside the computational array (including the propagation of errors through the POVM correction), as discussed above. The reported error bars on fit parameters are the standard error from the weighted least squares optimization fitting routine.

### Bounding the cross-talk of drives on neighboring qubits

**Fig. 6:**
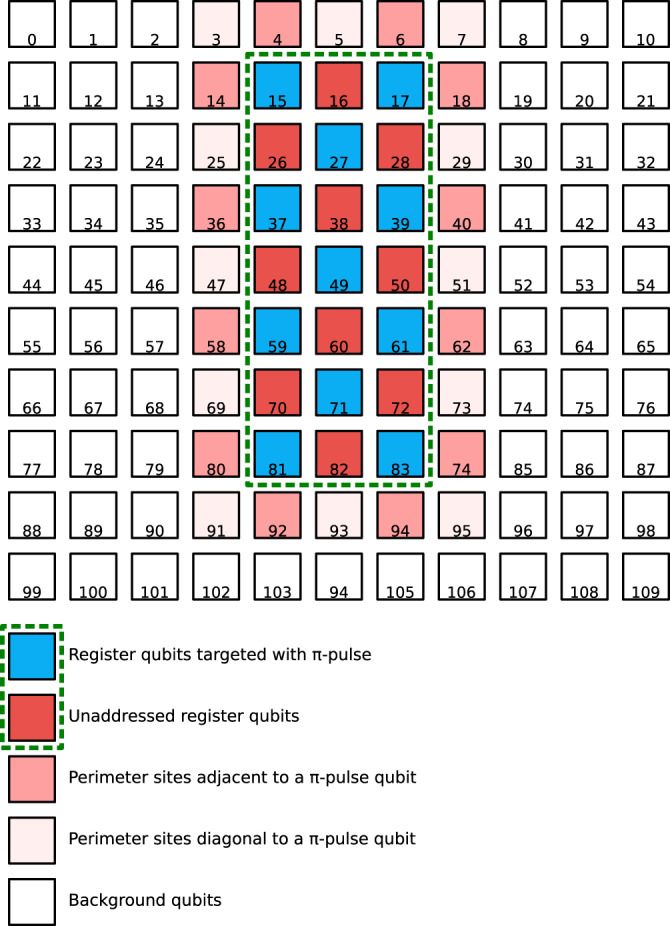
Diagram depicting schematically each site in the full 10 × 11 array and indicating the location of the computational array (green dashed box). The color of each box represents indicates qubits that are driven (blue) or undriven (red) within the computational array for the data presented in Fig. 3a. Additionally, the shading of the sites around the perimeter of the computational array indicates whether that site neighbored a driven qubit or an undriven qubit. All qubits more than one site away from the computational array are categorized as background atoms and are assumed to be undriven by the gate beams.

As we are driving each qubit individually, it is important to consider the cross-talk of the drive lasers from one site driving rotations of its neighboring atoms. In this context, a qubit is isolated from its neighbors’ drive beams if performing a state-changing operation on one qubit does not result in its neighbors being simultaneously driven to change state. While further characterization of this cross-talk with dedicated experiments will be required for demonstrating high-fidelity operations, we can use the data presented in Fig. 3a to estimate a bound on the cross-talk between sites because we have driven *π*-pulses on atoms in a checkerboard pattern within the computational array. Specifically, we compare the population of the qubits in the computational array that are undriven (and thus neighboring a driven qubit, depicted as red boxes in Fig. 6) to those that are not neighboring the computational array (white boxes in Fig. 6) and attribute the difference entirely to cross-talk.

We do not include signal from trap sites immediately neighboring the array in this analysis because the statistics are quite low (those sites will be rearranged into the computational array first). For reference, there is not a significant difference between the populations in atoms neighboring, $\left(0.110\pm 0.008\right)$, versus diagonal to the driven atoms, $\left(0.122\pm 0.005\right)$, along the perimeter of the computational array (and each of those two populations are within two sigma of the background populations).

The population of the undriven qubits in the computational array is $\left(0.128\pm 0.002\right)$ and the background atom population is $\left(0.113\pm 0.001\right)$, leading to a significant difference in population of $\left(0.015\pm 0.003\right)$. This 1.5% level difference, when fully attributed to cross-talk, implies a bound on the average cross-talk to neighboring qubits of $\left(0.005\pm 0.001\right)$, based on their being on average 2.9 driven neighbors to each undriven atom in the computational array.

If the beam were a perfect gaussian, this would be consistent with a larger-than-expected waist of ~2.4 μm, which could indicate a mismatch in the focal plane of the drive beams with respect to the trapping potentials (although to fully explain this discrepancy, the mismatch would need to be on the order of 10 μm, which seems unlikely given our alignment references). However, we know the beams are apertured into the objective, which leads to the actual profile including rings around the central spot. Combining this effect with imperfections in the beam shape (due to, e.g., astigmatism introduced by the AODs), could explain this level of cross-talk. Future work will aim to fully characterize the cross-talk between neighboring qubits, as well as to modify the beam shaping optics to minimize the cross-talk between neighboring qubits, a requirement for realizing high-fidelity local qubit manipulations.

Furthermore, we emphasize that it is possible some of this population imbalance could stem from differences in the state preparation for atoms initialized in the computational array (roughly 50% of which were rearranged into the computational array) compared to those left outside the computational array. While the rearrangement process should not couple the nuclear spin states, the process can lead to heating of the atoms, which would degrade the shelving pulse fidelity, resulting in the increased signal^35^. Further studies will aim to determine how much of this population imbalance is due to cross-talk of the manipulation beams compared to different state preparation and shelving performance in the computational array.

## Supplementary information

Description of Supplementary Data Files

Dataset 2

Dataset 1

## Data Availability

The processed data used to create all figures in this article are included in Supplementary Data files. The raw data (i.e. camera images from which tweezer occupation/qubit state is read out), which were used to create this processed data, are available upon reasonable request to the corresponding authors.

## Code availability

No custom code was used that is central to the conclusions of the manuscript. The data analysis routines can be made available upon reasonable request to the corresponding authors.
