# Supplementary material for: Assembly and coherent control of a register of nuclear spin qubits
Source: Nat Commun. 2022 May 19;13:2779. doi: 10.1038/s41467-022-29977-z (PMC9120523; doi:10.1038/s41467-022-29977-z)
Supplement: Supplementary file 1 — Description of Supplementary Data Files [file 41467_2022_29977_MOESM1_ESM.pdf]

Description of Supplementary Data Files

File name: Supplementary Dataset 1
Description: This .xlsx file is a spreadsheet with multiple sheets, each containing the data used for producing plots present in the following manuscript figures:

- Sheet 1: Contains data used for the creation of Figure 1e
- Sheet 2: Contains data used for the creation of Figures 2c and 2d
- Sheet 3: Contains data used for the creation of Figures 3a and 3b
- Sheet 4: Contains data used for the creation of Figures 4a and 4b
- Sheet 5: Contains data used for the creation of Figure 5a, 5b, and 5c

File name: Supplementary Dataset 2
Description: This CSV file contains a list of the "Processed detector counts" used for creating the histogram in Figure 1d. The presence of non-integer values for "detector counts" is due to the fact that before summing pixels across a region of interest, we first multiply the image by a mask with weights proportional to the average number of photons detected at a given pixel. The masks are created by averaging together many images of atom arrays and normalizing the final result so that the brightness of all sites is equal. A constant offset is then subtracted so that the average value in regions where no photons fall is approximately zero, which is why some “detector counts” result in negative values.
